# Binge eating is associated with trait anxiety in Korean adolescent girls: a cross sectional study

**DOI:** 10.1186/s12905-017-0364-4

**Published:** 2017-01-21

**Authors:** Jin-Yi Jung, Kye-Hyun Kim, Hee-Yeon Woo, Dong-Won Shin, Young-Chul Shin, Kang-Seob Oh, Eun-Hee Shin, Se-Won Lim

**Affiliations:** 1 Department of Psychiatry, Kangbuk Samsung Hospital, Sungkyunkwan University School of Medicine, 29 Saemunan-ro, Jongno-gu Seoul, 03181 South Korea; 2 Department of Obstetrics and Gynecology, Kangbuk Samsung Hospital, Sungkyunkwan University School of Medicine, Seoul, South Korea; 3 Department of Laboratory Medicine, Kangbuk Samsung Hospital, Sungkyunkwan University School of Medicine, Seoul, South Korea; 4 Department of Nursing, Sangji University, Wonju, South Korea

**Keywords:** Binge eating, Stress, Anxiety, Depression, Adolescent

## Abstract

**Background:**

Binge eating occurs more frequently in women than in men, and is known to be related to psychological factors such as stress, depression, and anxiety. This study examined the relationship between binge eating and depression, trait anxiety, and perceived stress in Korean adolescents.

**Methods:**

Four hundred girls (aged 17–18 years) from two high schools located in Seoul completed self-report questionnaires. In total, 327 participants returned reliable responses, and were included in the final study. Binge eating was measured using the Bulimic Inventory Test Edinburgh. The questionnaire also included the Perceived Stress Scale (PSS), Trait Anxiety (TA) of State-Trait Anxiety Inventory, Anxiety Sensitivity Inventory (ASI), and Beck Depression Inventory (BDI).

**Results:**

The binge-eating group had higher BMI than the control group. The binge-eating group showed higher scores than control on the PSS, BDI, ASI, and TA. The TA was most highly correlated with binge eating. From logistic regression analysis, TA was revealed to be the only factor that raised the risk of binge eating, whereas PSS, BDI, and ASI showed no statistical significance.

**Conclusion:**

Although binge eating was correlated with perceived stress, depression, and trait anxiety, when their influences were controlled, only binge eating appeared to be associated with trait anxiety.

## Background

Adolescence is a stage of psychological development during which an individual’s identity and independence from parents becomes established, along with an increased sensitivity to physical developments. Eating disorder is known to commonly occur during late adolescence or early adulthood [1], and is related to the physical, psychological, and social maturation of adolescents. It is common for patients with eating disorders to have coexisting mental disorders, which include a high likelihood of depression, alcohol dependence, and other anxiety disorders [2].

Individuals with binge-eating disorder engage in recurrent binge-eating during which they eat an abnormally large amount of food over a short period. Binge-eating disorder is the most common eating disorder. Twelve-month prevalence of binge-eating disorder among U.S. adult (age ≥18 years) females and males is 1.6% and 0.8%, respectively. Life-time prevalence of anorexia nervosa and bulimia nervosa among Koreans is 0.01% and 0.1%, respectively [3]. However, no reliable studies exist addressing the prevalence of binge-eating disorder in a Korean population. Social and cultural influences are considered one of the major risk factors for binge eating [4]. Besides daily activity-related stress and social stress, binge eating is considered to be related to psychological issues, especially depression. Previous studies have reported that the majority of people who did binge eat showed a higher level of depression than normal healthy people [5]. It was also found that binge eating is triggered by events and problems that act as stressors [6]. Other studies on patients diagnosed with binge-eating disorder reported that they were also diagnosed with various forms of anxiety disorders [7], and they consumed more food when they experienced anxiety [8].

The aim of this study was to investigate the relationships between psychological factors (such as depression, anxiety, and stress) and binge eating behaviors in Korean adolescents. Our study participants were a group of female high school students.

Eating behaviors are not only affected by mental and psychological factors such as depression, anxiety, and stress, but can also be affected by external factors, such as social, cultural, and environmental [9]. Considering this, the participants were selected from among female high school students who attended the same school. Korean high school students from the same school are considered to share a strong emotional bond because they wear identical uniforms, eat the same school-provided lunch, and spend most of their time (from 08:00 to 17:00, 5 days a week) studying at school. By studying these female high school students, we intended to reduce the likelihood of the social, cultural and/or environmental external factors that may affect their eating behavior.

## Methods

### Participants

Participants were 400 female students selected from two adjacent women’s high schools in Seoul, Korea. To minimize the possible bias associated with difference in socioeconomic state among students, the authors chose two schools that are located only 2 km away from each other within the same district. Among those recruited, 350 students answered our questionnaire. A total of 23 participants were excluded from the analyses due to missing data, leaving a final total of 327 students.

In our study, the questionnaire was a self-reporting assessment, where the participants filled out their age, current weight, and height. Body mass index (BMI: weight (kg)/height (m^2^)) of the students was calculated from these data. The percentiles and z score values for BMI-for-age relative to the World Health Organization (WHO) 2007 reference were calculated using WHO AnthroPlus [10]. Overweight (> + 1SD BMI-for-age z score), obesity (> + 2SD BMI-for-age z score), thinness (< −2SD of BMI-for-age z score), and severe thinness (< −3SD of weight-for-age z score) were defined according to the WHO references [11].

### Ethical considerations

The Kangbuk Samsung Hospital Institutional Review Board (IRB) reviewed and approved the study. The Kangbuk Samsung Hospital IRB was officially certified by the 2009 The Forum for Ethical Review Committees in Asia and the Western Pacific Region. The researchers of this study had visited the schools, explained the aim of the study and guaranteed anonymity. All participants provided informed consent prior to enrollment. Because the participating students were underage, the study was started only after receiving the completed consent form from their parents.

### Measurement

Assessment tools used for the questionnaire were as follows:

#### Bulimic investigatory test Edinburgh (BITE)

This is a scale designed by Henderson and Freeman [12]. It consists of 33 questions and is designed to diagnose binge eating or bulimic conditions, and to determine their severities. The maximum score of symptom subscale is 30: >20 indicates the existence of an eating disorder and binge eating; 10–19 indicates the existence of an unusual eating behavior; a severity subscale >5 is seen as clinically significant. In our study, we determined the binge-eating group as the one where the score is >10, because low scorers with a score of below 10 indicates the absence of both compulsive eating and binge eating, and scorers with a score of 10 or more suggests an unusual eating pattern [12]. Although the Korean version of BITE was not standardized, it was studied in the adolescent population in a previous Korean study, and this scale showed moderate reliability (Cronbach’s α = 0.7587) [13].

#### Perceived stress scale (PSS)

The PSS is a self-reporting scale designed by Cohen and colleagues [14]. It consists of 14 questions in total, with answers based on the occurrences of the past 3 months. It takes approximately 3 min for the patient to complete, and no special education or training is required to administer it. The total score is calculated by adding all item scores. In general, we found that women’s scores were 1 or 2 points higher than men’s scores. The Korean version of the PSS was previously shown to have an internal consistency of 0.82 [15]. The overall Cronbach’s α was 0.819, and the test-retest reliability coefficient was 0.66 [16].

#### Korean version of beck depression inventory (BDI)

The BDI is a self-reporting questionnaire consisting of 21 questions, addressing emotional, cognitive, synchronous and physiological symptoms. Items are scored along a 4-point scale, from 0 to 3, with a total score in the range of 0 to 63. Beck classified a score <9 as non-depression; 10–15 as mild depression; 16–23 as moderate depression, and ≥24 as severe depression [17]. Participants were asked to complete the validated Korean translated versions of the BDI [18–20]. Lee and Song standardized the Korean version of the BDI, and the reported psychometric properties was good (Cronbach’s α 0.93, test-retest reliability coefficient r = 0.91, consistency coefficient = 0.85) [21].

#### Anxiety sensitivity inventory (ASI)

Our study used the Korean-targeted anxiety sensitivity scale, which was standardized by Kim and colleagues, to customize for a Korean population (Cronbach’s alpha = 0.93) [22]. It is a self-reporting test measuring the tendency of experiencing “anxiety for anxiety” and anxiety sensitivity, which is an excessive and sustained response to an anxiety-triggering stimulus. The total score range is 0–64. For the Korean-targeted study [23], the sensitivity response to anxiety stimuli is classified as follows: 16–20 = somewhat sensitive; 21–24 = significantly sensitive; ≥25 = very sensitive. Three subfactors are addressed: fear of symptoms from respiratory, cardiovascular and gastrointestinal systems (physical anxiety), fear of being evaluated by others (social anxiety), and fear of uncontrollable cognitive aspects (psychological anxiety).

#### State-trait anxiety inventory (STAI)

The STAI is a self-reporting assessment for measuring anxiety, developed by Spielberger [24]. We used the Korean version of STAI adapted by Kim [25], which has a Cronbach α reliability coefficient of 0.89 (trait anxiety) and 0.93 (state anxiety). Each section consists of 20 questions, totaling 40 questions. Although state anxiety is a subjective emotional state that can frequently change, its severity is dependent on the situation and time. Trait anxiety is a behavioral tendency that is constant, thus making it important to be measured and analyzed for our study. Previous studies have shown conflicting correlation between binge eating and anxiety [26, 27]. One of the goals in the present study was to examine the relationship between binge eating and the type and levels of incessant stress, not only temporary conditions of worries and tension. Therefore, only trait anxiety was measured. Trait anxiety is regarded as a characteristic of an individual’s personality particularly associated with binge eating behavior.

### Statistical analysis

The author categorized the participants into two groups: if an individual’s BITE was >10, they were included in the binge-eating group, and if their BITE was <10, they were included in the control group [12]. Following this, independent variable *t*-test was used for comparing and analyzing BMI, stress, trait anxiety and depression between the two groups. Correlation analysis was done to examine the relationship between binge eating and stress, trait anxiety, and depression. Binomial logistic regression analysis was done to investigate the risk level of stress, depression, and trait anxiety on binge eating. The significance level of each statistical analysis was determined as *p* < 0.05. The data collected in our study was statistically processed using SPSS version 18.0 (IBM Co., New York, NY, USA).

## Results

The final 327 participants included in the survey assessment were categorized into the binge-eating and control groups. Overall, the participants’ mean age was 18.0 years, and their mean menarcheal age was 13.4 years. Although the binge-eating group had an older mean age and earlier menarcheal age than the control group, it was not statistically significant (*p* = 0.051 and *p* = 0.098, respectively).

The participants’ mean BMI percentile and z-score were 43.34 ± 25.9 and −0.21 ± 0.8, respectively; the binge-eating group had higher BMI than the control group (t = −4.293, df = 325, *p* < 0.000). The binge-eating group had a higher PSS than the control group (t = −3.190, df = 325, *p* = 0.002). The binge-eating group had higher BDI than the control group (t = −5.300, df = 325, *p* < 0.000). The binge-eating group showed statistically significantly higher ASI than control group. (t = −3.821. df = 144.304, *p* < 0.000). The binge-eating group had higher TA than the control group (t = −6.303, df = 325, *p* < 0.000) (Table 1).

**Table 1:** Demographic characteristics of study participants

	Total (*N* = 327)		Binge-eating group (*N* = 92)		Control group (*N* = 235)		*p*
	Mean	SD	Mean	SD	Mean	SD	
Age (years)	18.0	0.7	18.2	0.7	17.9	0.7	.051
Menstrual age (years)	13.4	2.6	13.3	1.2	13.5	1.2	.098
BMI (kg/m^2^)	20.84	2.6	21.79	2.8	20.46	2.4	<.001
BMI (percentile)	43.34	25.9	52.65	25.9	39.7	24.6	<.001
BMI (z-score)	-0.21	0.8	0.1	0.8	-0.33	0.8	<.001
PSS	16.13	4.5	17.38	4.9	15.63	4.2	.002
BDI	12.89	8.2	16.58	8.4	11.44	7.7	<.001
ASI	15.94	9.8	19.42	10.8	14.57	9.1	<.001
ASI_physical	6.40	5.4	7.85	5.9	5.83	5.1	.004
ASI_mental	4.63	4.0	6.22	4.7	4.01	3.5	<.001
ASI_social	5.26	2.3	5.65	2.0	5.11	2.4	.041
TA	50.06	9.3	54.97	9.0	48.14	8.7	<.001

*PSS* Perceived Stress Scale, *BDI* Beck Depression Inventory, *ASI* Anxiety Sensitivity Inventory, *TA* Trait Anxiety

To discover the explanatory factors for binge eating, we studied the correlation between the BITE score (which is a binge eating scale) and stress, depression, trait anxiety, and BMI. The results from a simple correlation analysis showed a quantitative correlation between binge eating and all of the aforementioned factors. Among them, trait anxiety showed the highest correlation with binge eating (r = 0.354, *p* < 0.001) (Table 2).

**Table 2:** Pearson’s correlation coefficient for BITE, symptom scale and BMI

	BITE	PSS	BDI	ASI	TA	BMI
BITE						
PSS	.279^a^					
BDI	.349^a^	.320^a^				
ASI	.303^a^	.375^a^	.546^a^			
TA	.354^a^	.371^a^	.689^a^	.571^a^		
BMI	.238^a^	.063	.036	-.020	-.003	

*BITE* Bulimic Inventory Test Edinburgh, *PSS* Perceived Stress Scale, *BDI* Beck Depression Inventory, *ASI* Anxiety Sensitivity Inventory, *TA* Trait Anxiety, *BMI* Body mass index

^a^Correlation significant at 0.01(bilateral)

The variables that had a statistically significant correlation from binominal logistic regression analysis were PSS (OR = 1.03, 95% CI = 0.97–1.10), BDI (OR = 1.02, 95% CI = 0.98–1.07), ASI (OR = 1.00, 95% CI = 0.97–1.04), and TA (OR = 1.07, 95% CI = 1.02–1.16). From the results of the binominal logistic regression analysis, using the four variables that were significantly related to binge eating, the variable that was included in the final regression equation was TA. The PSS, BDI, and ASI showed no statistically significant risks (Table 3).

**Table 3:** Logistic regression analysis demonstrating the contribution of stress, depression, and trait anxiety to binge eating behavior

	Coefficient	Odds ratio	95% CI of OR	*p*
PSS	.030	1.03	0.97-1.10	.362
BDI	.022	1.02	0.98-1.07	.304
ASI	.003	1.00	0.97-1.04	.837
TA	.066	1.07	1.02-1.12	.002

*OR* odds ratio, *CI* Confidence interval

*PSS* Perceived Stress Scale, *BDI* Beck Depression Inventory, *ASI* Anxiety Sensitivity Inventory, *TA* Trait Anxiety

## Discussion

From this study, 28% (*n* = 92) of students showed binge-eating behavior with a BITE (Bulimic Investigatory Test Edinburgh) score of >10. The binge-eating group had a significantly higher mean BMI than the control group.

A previous study on the correlation between eating behavior and emotional characteristics reported that depression and self-esteem were the main factors resulting in binge eating, with high levels of depression found in most participants who resorted to binge eating. Another study also reported that binge eating or eating suppression were outward behaviors reflecting participants’ desires to control negative emotions such as depression or anxiety [28]. Studies have also claimed that eating disorders occur alongside affective disorders, with affective disorders even increasing the risk of developing an eating disorder [29].

In our study, the higher BITE scores were associated with higher levels of stress, trait anxiety, and depression in the binge-eating group than that in the control. But when the influences were adjusted against each other, the high levels of trait anxiety were found to be associated with the risk of binge eating, but no significant differences were found for stress and depression. These findings are inconsistent with those of previous studies, which showed a high correlation between depression and binge eating [30]. The mean BDI score of all study participants was 12.89, meaning that most of the participants did not suffer from serious depression [31]. Although previous studies showed a high correlation between depression and binge eating when evaluated in patients with mood and eating disorders [32], our study group consisted of a normal population; thus possibly explaining the differing results when compared with previous studies.

A study examining mental disorder coexisting with eating disorders, bulimia nervosa and binge eating showed a higher risk of comorbidity for anxiety disorder, rather than anorexia nervosa [33]. The comorbidity between anxiety disorder and eating disorder was examined in another study. This investigation revealed that when the participants diagnosed with eating disorders were categorized into an anorexia nervosa group, anorexia nervosa/binge-eating disorder (co-occurring) group, and bulimia nervosa group, the ratio of having one or more co-occurring disorders from anxiety were 55%, 62%, and 68%, respectively [34]. These results show that if the eating disorder has a higher binge-eating behavior, there is a higher frequency of the onset of anxiety disorder, which is consistent with our finding of a significant difference between onset of binge-eating behavior and increasing anxiety. Contrary to studies indicating that anxiety associates binge eating, many studies have shown evidence that binge eating decreases anxiety. One study suggests that in the presence of an uncontrollable, threatening issue, concentrating on binge eating acts as a process of transferring the negative emotion to the food, which is under one’s control [35]. A large survey of Korean adolescents reported a high prevalence of interpersonal sensitivity (74.3%), depression (56.9%), and anxiety (48.8%), with girls scoring higher than boys in all three symptoms [36].

Our study measured levels of binge eating in female high school students using BITE (Bulimic Investigatory Test Edinburgh), and reviewed its relationship with stress, depression and anxiety. We demonstrated that anxiety, among the variables that affect binge eating, plays a more significant role than stress or depression. Trait anxiety is a general and long-standing quality of stress that is characteristic of an individual’s personality. Some people experience a greater appetite than usual when they are anxious, while others experience much less hunger and thirst with anxiety. This difference appears to be associated with the level of trait anxiety. As observed in this study, adolescents with high trait anxiety tend to binge eat. Because food and eating behavior could act as a coping mechanism to stress [37], high trait anxiety may lead them to craving food when they are suffering from anxiety. We propose public health authorities screen trait anxiety of adolescent students, and provide school or community-based educational programs about healthy eating and proper coping strategies to reduce anxiety. This could diminish the likelihood of adolescents at high risk for developing various kinds of eating disorder from actually doing so. Previous research has already suggested that the primary prevention programs should be offered early enough to prevent the onset of these potentially harmful behaviors [38]. Therefore, the adolescent binge eating might be prevented by educating schools and communities about the association between trait anxiety and binge eating, and by screening adolescents for mental health including trait anxiety.

Certain study limitations should be noted. First, this study was limited to two girls’ high schools; thus, our results cannot be considered as representative of the eating behavior of the entire female population of Korean high school students. Second, because we used a self-reporting questionnaire, the reliability of the data should be appraised.

Considering the prevalence of eating disorder is much higher in women, a great advantage of this study was that we could investigate eating behavior in a homogenous group of female adolescents, controlling for problematic factors such as sex and age. Culture-specific stressors such as excessive expectations and demands of parents for academic achievement, and peer conflict or rejection might contribute to psychological symptoms in Korean adolescents. Adolescents are more likely to be exposed to a variety of mental disorders due to frustration and increased aggressiveness caused by academic stress. Korean adolescents spent most of their time in school studying. Although sleep is essential for mental and physical health, Korean adolescents do not have enough time even for sleep. Seoul metropolitan government statistics reported that high school students in Seoul only sleep an average of approximately 5.5 h each night, going to bed around 01:00 and waking up at 06:30. This is far shorter than American Academy of Pediatrics’ recommendations (8.5–9.5 h of sleep per night) for adolescents [39]. Korean adolescents also have little time for leisure or sports activities, which can relieve stress. It has been reported that short sleep durations were associated with increased food intake in European adolescents [40]. Therefore, future studies should focus on the correlation between study-related stress, sleep deprivation and binge eating, because study-related stress to enter colleges is much higher in Korea than in other countries [41]. A prospective study for a designated time period might also reveal the cause and effect dynamics between binge eating and emotional factors. An accurate assessment of height, weight, and binge eating using semi-structured interviews by experts would be necessary.

## Conclusions

This study examined female adolescents to understand the correlation between the frequency of binge eating, and the emotional characteristics related to binge eating such as stress, depression, and anxiety. From this study, 92 students (28%) from a total 327 female high school students, showed a tendency to binge eat, having a quantitative correlation with BMI, PSS, BDI, ASI, and TA. The binge-eating group showed higher BMI, stress, depression, and anxiety than the control group. However, when the influence of these factors were adjusted against each other, the higher level of trait anxiety was associated with the risk of binge eating, but there was no significant correlation between binge eating, stress, and depression.
